# Stakeholders’ opinions on the implementation of Child Death Review in the Netherlands

**DOI:** 10.1186/s13104-016-1966-x

**Published:** 2016-04-21

**Authors:** Sandra Gijzen, Monique P. L’Hoir, Magda M. Boere-Boonekamp, Ariana Need

**Affiliations:** Department HTSR, IGS Institute for Innovation and Governance Studies, University of Twente, PO Box 217, 7500 AE Enschede, The Netherlands; TNO Child Health, P.O. Box 2215, 2301 CE Leiden, The Netherlands; Department Public Administration, IGS Institute for Innovation and Governance Studies, University of Twente, PO Box 217, 7500 AE Enschede, The Netherlands

**Keywords:** Implementation, Child mortality, Child Death Review, Prevention

## Abstract

**Background:**

The death of a child is an enormous tragedy for both the family and others involved. A child’s death appeals to everyone’s responsibility to take measures to prevent similar deaths in the future. Child Death Review (CDR) is an interagency approach in which a child’s death is systematically analyzed by a multidisciplinary team. The aim of CDR is to identify avoidable factors that give direction to prevention and to improve death statistics. CDR is not yet implemented in the Netherlands. The purpose of this study is to determine Dutch stakeholders’ opinions regarding the facilitating and impeding factors in the implementation of CDR in the Netherlands.

**Methods:**

Four focus groups were conducted: three with professionals who are involved in children’s deaths and one with parents who have lost a child under the age of 2 years. The recorded discussions were transcribed and analyzed using Atlas ti. The facilitating and impeding factors were measured using the measurement instrument for determinants of innovations (MIDI). The MIDI identifies facilitating and impeding determinants associated with the innovation, user, organization and social-political context.

**Results:**

Improvement of the quality of (health) care and obtaining a clear explanation for the child’s death (*user and innovation*) were identified as benefits of CDR. The emotional burden for professionals and parents and the time implications were considered to be drawbacks of CDR (*user and innovation*). The multidisciplinary approach (*innovation*), parental consent and the use of anonimyzed data (user) were considered as facilitators to implementation. Insufficient information (*innovation*), potential legal consequences for professionals and organizations (*user*), insufficient ratification by organizations (*organization*) and confidentiality (*social-political context*) were identified as impeding implementation.

**Conclusions:**

The determinants identified as facilitating and the recommendations provided to overcome the barriers can be used as input for the strategy for implementation of CDR. A pilot study is necessary to determine to what extent the chosen implementation strategy is effective.

**Electronic supplementary material:**

The online version of this article (doi:10.1186/s13104-016-1966-x) contains supplementary material, which is available to authorized users.

## Background

Children are expected to grow into adulthood in a safe and healthy environment. It is the responsibility of every state to promote the welfare of children and protect them from harm [1, 2]. When a child dies, it is a great tragedy for both the family and relatives, friends, neighbors, and other acquaintances [3, 4].

Concerted efforts remain necessary to avoid child deaths in the coming years and to accelerate further progress in improving child survival [5].

In the United States of America (USA), Canada, Australia, New Zealand and the United Kingdom (UK), a structured approach is being used where different agencies work together in order to understand why children die and how future deaths can be prevented. This approach is called Child Death Review (CDR). The objectives of CDR are to identify avoidable factors in child deaths, to translate the results in recommendations which may prevent future deaths and improve child health and welfare, and to improve cause of death statistics [6, 7] and the support to the family [8]. In the USA and UK, CDR consists of two interrelated parts: a rapid response investigation in cases of unexpected deaths and a retrospective panel review of all child deaths by a multidisciplinary team. This multidisciplinary team consists of core members [6, 7, 9]. Studies have shown that CDR has identified modifiable factors in child deaths [7, 8, 10, 11]. Implementation of recommendations based on the CDR method locally, regionally and nationally have resulted in the prevention of child deaths [7, 12].

In the Netherlands CDR is not implemented yet. If the CDR were to be introduced, it would require support and involvement of the parents of a deceased child and professionals in child and family (health) care. A bottom-up approach is desirable in developing an implementation strategy, because it may increase the motivation of professionals to integrate the CDR-procedure in their own (clinical) practice [13]. For the successful implementation of an innovation in current (health) care structures, ideally, all stages of the process of change, i.e., dissemination, adoption, implementation and continuation, should be passed. At each stage different factors may facilitate or impede the process of change [13, 14]. It is important to get insight in which impeding and facilitating factors might influence the different stages of the implementation of CDR in the Netherlands.

In this study, started from February 2010 till January 2011, we examined the opinions of stakeholders about the implementation of CDR in the Netherlands. We focused on the creation of support among stakeholders (adoption), the actual implementation and securing CDR in existing practice (continuation). Important stakeholders were asked for their opinions regarding the facilitating and impeding factors in the implementation of CDR. The research question of this study is twofold: (1) what are the stakeholders’ opinions regarding the facilitating and impeding factors in the implementation of CDR in the Netherlands; and (2) which recommendations do stakeholders give for the implementation of CDR in the Netherlands? We planned to use the results of this study to design a pilot implementation.

## Methods

### Study design

To answer our research question we used a qualitative, descriptive design. We held focus group discussions to identify stakeholders’ opinions regarding facilitating and impeding factors in the implementation of CDR in the Netherlands. We used the measurement instrument for determinants of innovations (MIDI), developed by Fleuren et al. [14]. This is a qualitative coding framework consisting of four domains, each of which contains a number of determinants associated with the innovation; with the adopting person (user); with the organisation; and with the socio-political context. A description of all 29 determinants [15] is provided in Additional file 1: Appendix S1. The MIDI identifies facilitating and impeding factors in the four domains [14]. We consider CDR as an innovation because it concerns a working method that is new to the Netherlands, [16] even though it has been implemented elsewhere previously. According to the criteria of Dutch Medical Research Involving Human Subjects Act, this study did not need to be submitted for ethical approval by a Medical Ethical Committee [17]. Therefore the study was reviewed by the institutional Ethical Committee of the University of Twente (Ethical Committee (EC) of the faculty of Behavioral Sciences, reference number 16039) and approved.

### Study sample

The target group of stakeholders consisted of professionals who are directly or indirectly involved in a child’s death and parents who have lost a child under the age of 2. Because the results of this study were going to be used for a pilot implementation, we recruited professionals as representatives of healthcare organizations located in the pilot region (two eastern provinces of the Netherlands). We recruited the professionals through their managers or head of the departments of the health and child care organizations where the professionals work. Parents were recruited through the boards of the Parents’ Association of Cot Death Children (in Dutch: *Vereniging Ouders van Wiegedoodkinderen*) [18] and of the Parents’ Association of a Deceased Child (in Dutch: *Vereniging van Ouders van een Overleden Kind*) as representatives of these associations [19]. Professionals and parents were invited to participate in the focus groups by means of an invitation letter, which was sent by regular mail. The invitation letter contained information about the objective of the study and a short description of the method used. Twenty-one professionals and four parents signed up by email.

### Data collection

Data were gathered through four face-to-face focus group discussions in May and September 2010. Of the 21 professionals who signed up, sixteen are professionally involved in the care for the child and his family at the very moment when a child dies and five in the period afterwards. These professionals were divided in three focus groups, as shown in Table 1. The fourth focus group consisted of three parents of a deceased child. The fourth parent who signed up was unable to join the focus group discussion (see Table 1).

**Table 1:** Number and background of participants in each focus group

Participating professionals/parents	Focus group 1	Focus group 2	Focus group 3	Focus group 4
	N	N	N	N
Pediatrician	3	4		
General practitioner	1	1		
Forensic physician	1	1		
Preventive child health CARE professional (physician/nurse)	1	2		
Social worker		2		
Physician of the child protection service			1	
Police officer			1	
Mental health care physician			2	
Manager of organization that provides support to children and adults and their families with disabilities (MEE)			1	
Parents				3

Each focus group was moderated by the second or third author. The first author took notes and audiotaped each session with consent of the participants. An agenda and a semi-structured interview schedule were used to guide the focus group discussions. In total six questions were asked in each focus group about the participants’ opinions of the (dis)advantages of CDR (1) in general, (2) for the parents, (3) for professionals who provide information or (4) for participants in the CDR team, (5) for organisations, and (6) when CDR is implemented at a national level. The audiotaped focus group discussions were transcribed by the first author and the data were anonimyzed. The four transcribed records were used for analysis.

### Data analysis

The transcribed records were analyzed using Atlas ti. [20] A codebook was created based on the determinants of the MIDI [15]. A determinant was judged to be a facilitator if a participant described it in a way which indicated that it would lead to or enhance the achievement of the objectives of CDR or its implementation (i.e., positive labelling of a determinant). When a determinant was described by a participant in a way which indicated that it would hinder or reduce the achievement of the objectives of CDR or its implementation, it was judged as impeding (i.e., negative labelling of a determinant). Relevant text fragments corresponding to the determinants of the MIDI were selected by a second coder (master student). Next, the first author coded all online focus groups and the second coder coded the same focus groups independent from the first author to minimize bias introduced into the results by researchers’ selection. Subsequently, the text fragments that were provided with codes by the two coders were compared with each other. All differences in coding were discussed between the coders. Ultimately, consensus was reached about the definitive set of codes and the text fragments that corresponded to these codes. The codes with corresponding text fragments were arranged in order of the determinants of the MIDI. A subdivision was made into the facilitating and impeding determinants of the CDR method and of effective implementation, and stakeholders’ recommendations.

## Results

Thirteen of the 29 determinants presented by Fleuren et al. [15]. were identified by the stakeholders as facilitating or impeding. Six of these determinants were mentioned in all four focus groups. Most of determinants were identified in the category “innovation” and “user”. We did not find facilitating or impeding determinants that were not mentioned in the MIDI. The determinants identified in the focus groups are presented in Table 2.

**Table 2:** Number of times participants in the focus groups mentioned MIDI determinants [15] as facilitating or impeding in het implementation of CDR

Determinants	Facilitating	Impeding
Determinants associated with the characteristics of the innovation		
Procedural clarity	1	2
Completeness	6	18
Complexity	–	1
Compatibility	1	–
Relevance for client	12	5
Determinants associated with the characteristics of the adopting person (user)		
Personal benefits/drawbacks	14	25
Outcome expectations	29	9
Client cooperation	18	23
Descriptive norm	1	1
Knowledge	–	3
Determinants associated with the characteristics of the organisation		
Formal ratification by management	1	1
Time available	–	7
Determinants associated with the socio-political context		
Legislation and regulations	8	9

In the presentation of the results we first focus on the benefits and drawbacks of CDR as a method and subsequently on the facilitators and barriers to effective implementation. The determinants are summarized and illustrated with relevant quotes. All quotes from professionals and parents regarding the facilitating and impeding determinants are presented in Additional file 2: Appendix S2.

### Benefits of CDR

Benefits of CDR were identified in personal benefits and outcome expectations (determinants associated with the user). With regard to *personal benefits* three benefits were mentioned directed at professionals. First of all participants perceived CDR as an instrument to check whether or not the professional responded to the death according to established guidelines and/or protocols. A second benefit is indicated in the following quote:

*“Positive is the fact that you are immediately aware of reporting every child death to the Child Death Review team, and that you have to report every death, which is not an automatic procedure.”* (focus group: professionals)

Third, professionals might also benefit from the findings of the CDR team. Reviews can enable improvement of the quality of (health) care and the education of professionals. With regard to parents two benefits were mentioned by participants. First, it was noticed that CDR might provide parents a clear explanation for their child’s death, which can be considered as a second opinion. Second, well conducted death reviews might influence the mourning process of parents positively. Support of the family was perceived as important. With respect to *outcome expectations*, participants first of all expected that through conducting reviews, substandard factors in (health) care could be identified which could lead to recommendations from different perspectives in order to improve (terminal) care. Second, they experienced an added value in aggregating data to identify certain patterns in child deaths from which recommendations might be translated into regional or national policy to prevent future deaths. Third, it was expected that reviewing a child’s death might result in a better classification of the death, for example in cases of unrecognized child abuse.

The relevance for the client (i.e., professionals and parents), a determinant associated with the innovation, was identified as another benefit of CDR. Participants considered the understanding of the circumstances leading to death as *relevant* for professionals and parents. It was mentioned that professionals might learn from each other. They also might use the conclusions of the CDR team for their own practice in order to improve the quality of care. With regard to parents, analyzing a child’s death was perceived as showing respect to the child. The following quote indicates a second relevance for parents:

“*A strength of the method is the fact that a review is not only conducted in cases of special circumstances, but in every child death. CDR is offered to every parent of a deceased child. So, it is not assumed that suspicious circumstances had been present leading to death*.” (focus group: professionals)

In the focus groups it was also mentioned that CDR might be an added value in the identification of specific groups of child deaths.

There were no determinants associated with the organisation and socio-political context identified as benefits of CDR.

### Drawbacks of CDR

The emotional burden for professionals and parents was perceived as a drawback associated with *personal benefits* (determinant associated with the user) and the *relevance* for the parents (determinant associated with the innovation). Next to this, some of the participants indicated that it is time consuming to provide information, to anonymize data and to coordinate everything in order to review a child’s death. In relation to *outcome expectations* (determinant associated with the user) and *relevance* for the parents (determinant associated with the innovation) participants discussed whether CDR has an added value in individual cases and deaths due to natural causes. Next to this, it was expected that parents do not want to sign the consent form shortly after the death of their child or when consent is asked by an unknown person. Chemistry in contact was mentioned as important. Another reason not be willing to sign the consent form is illustrated in the following quote:

“*I think that if parental consent for autopsy is asked in a blunt manner or by a wrong person at the wrong time and as a parent you have said ‘no’, then this will determine the further course of the investigation*.” (focus group: parents)

There were no determinants associated with the organisation and socio-political context identified as drawback of CDR.

### Facilitators to effective implementation

Procedural clarity, completeness and compatibility (determinants associated with the innovation) were identified in the focus groups as facilitating to effective implementation. With respect to the *procedural clarity* it was considered as important to know which professional meets the parents in order to obtain their consent. In relation to *completeness* of the CDR method, three facilitating factors were identified. First, the multidisciplinary approach was mentioned as facilitating as illustrated in the following quote:

“*If all disciplines provide information, the chance will increase to come to the proper reconstruction about what exactly happened. When you hear that from one perspective, it is always coloured and contains miscommunication and occupational deformation.”* (focus group: parents)

Second, the fact that feedback of the findings is provided to professionals was considered as a facilitating factor. Third, the presence of a behavioural scientist as one of the core members of the CDR team was perceived as positive as illustrated in the following quote:

“*With regard to the evaluation of the aftercare I think the presence of a behavioural scientist as one of the core members of the CDR team is positive*.” (focus group: professionals)

In relation to *compatibility*, similarities with the audits of perinatal deaths, that are common practice in the Netherlands, were identified as facilitating.

The cooperation of the client and descriptive norms that are determinants associated with the user were other facilitators identified in relation to effective implementation. Regarding the *cooperation of the client*, participants expected parents to cooperate more easily when they are fully informed about the objective and procedure of CDR shortly after the death of their child and are asked to provide consent in written form a few weeks after their child’s death. It was mentioned that signing a consent form emphasizes the respect towards the parents who have lost their child. Next to the informed consent a second facilitating factor in the cooperation of parents is illustrated in the following quote:

*“As a parent you have lost a child that is very special, but at the same time you can do something positive. By cooperating in CDR parents could contribute to the prevention of future deaths.”* (focus group: parents)

In order to obtain cooperation of professionals the use of anonymized data to analyse the causes of child deaths was mentioned as a benefit. In relation to descriptive norms some participants expected that only a few of their colleagues would participate in CDR, because participation is assumed to be an emotional burden and time consuming.

There were no facilitators identified in relation to the determinants associated with the organisation.

With regard to the determinants associated with the socio-political context the following quote illustrates what was identified as facilitating to implementation:

“*If parents* gave their consent, confidentiality is not a problem anymore.” (focus group: professionals)

### Barriers to effective implementation

Procedural clarity, completeness and complexity (determinants associated with the innovation) were identified as barriers to effective implementation. In relation to the *procedural clarity* it was mentioned that professionals might decide not to notify a child’s death to be reviewed when clear agreements about feedback to professionals are lacking. Next to this, the stratification of the CDR process was perceived as unclear. To gather all information from the (medical) files of the deceased child necessary to analyze the death properly was considered as troublesome and therefore as a barrier related to *completeness* of the CDR method. First of all, as noticed in the following quote:

“*Not everyone has an extensive (medical) file, but one can have a lot of experience with the parents or deceased child that is not noted in the (medical) file*.” (focus group: professionals)

Second, as indicated in one focus group data in electronic files can be changed, which was perceived as worrisome. Third, it was mentioned that information systems used in organizations within and between regions differ from each other which might hinder the exchange of information. In response to this, participants discussed the fact that professionals might decide not to provide information, despite the presence of parental consent. This would certainly concern cases in which the Public Prosecutor is investigating the death. Another barrier related to *completeness* of the CDR method is illustrated in the following quote:

“*What surprises me a bit is that professionals involved are not present during the CDR meeting. I think that’s remarkable, because that will make the communication more equally clear and obvious. Written information could be misinterpreted*.” (focus group: professionals)

Regarding the *complexity* it was mentioned that it requires a lot of energy for professionals to find out in detail the circumstances leading to death.

The cooperation of the client, descriptive norm and knowledge that are determinants associated with the user were identified as other barriers to effective implementation. The legal consequences for professionals and organizations was perceived as a barrier for *cooperation*. Despite the fact that the CDR team will analyse a death with all due care without blaming someone, parents might sue professionals if they know that substandard factors in care have contributed to their child’s death. Not only professionals but also parents might be anxious to be considered partly responsible for the death. Therefore, they might decide not to participate in CDR especially when a child’s death is expected to result in negative publicity in the media. Another barrier identified is indicated in the following quote:

“*If you are involved in such a case, especially if you’re directly involved, it will cost you emotionally and practically very much time. Then the paperwork is not that what everyone is waiting for*.” (focus group: professionals)

The fact that professionals might perceive participation to CDR as time consuming was considered as another barrier. Finally, parents might decide not to participate when they perceive that they will not get full disclosure of the findings of the CDR team. Participants wondered whether CDR team members have sufficient *knowledge* to analyse medical procedures. In relation to the *descriptive norm* it was mentioned that it might be difficult to obtain the cooperation of all paediatricians.

With regard to the barriers associated with the organisation it was indicated that professionals who are requested to provide information to the CDR team have busy work schedules and not enough time. Another barrier identified is illustrated in the following quote:

“*When actions should be set out within certain professional groups that are employed, you have to do with a management that must support these actions and has to give time to be able to implement them in practice*.” (focus group: professionals/parents)

Of the determinants associated with the socio-political context barriers were found in the professional confidentiality, the involvement of the Public Prosecutor and the Dutch rules and regulations as indicated in the following two quotes:

“*In the interest of the investigation which is still ongoing, a forensic physician just can’t give information merely because he/she can only report to the Public Prosecuto*r.” (focus group: professionals)

“*It should be figured out how CDR fits well into the Dutch system of health care and justice that is a totally different culture in relation to other countries where CDR is implemented*.” (focus group: professionals)

### Recommendations of stakeholders

Both professionals and parents who participated in the focus group discussions provided recommendations. These are arranged according to the four groups of determinants and summarized in Table 3. Most recommendations are directed at determinants associated with the innovation, i.e., procedural clarity and completeness, and associated with the user, i.e., personal benefits and client cooperation. With regard to the innovation it was recommended that a format should be used to guide the conversation with parents in order to obtain their consent and to help professionals in providing information that is needed to review a child’s death. Second, the general practitioner, preventive child health care professional or paediatrician should be approached for information as a standard procedure. Third, in case the death of a child is investigated by the Public Prosecutor agreements should be made for reviewing the death. Fourth, CDR could join other review processes that are conducted in the Netherlands. Finally, feedback of the findings should be given to professionals and parents. In relation to the determinants associated with the user it was recommended that the CDR team should be independent and chaired by a person who has an overall view and is objective. Second, the time investment of the CDR team members should be clear. Third, in order to obtain the cooperation of parents they should be fully informed about the objective of CDR and should be asked for consent a couple of weeks after the death of their child. Finally, data should be anonymized at an early stage. With regard to the organisation and socio-political context time should be facilitated by managers and CDR should be adjusted to the Dutch law and regulations respectively.

**Table 3:** Recommendations provided by the professionals and parents who participated in the focus groups categorized in four groups of determinants: (1) innovation, (2) user, (3) organization. (4) socio-political context

Determinant	Recommendations
Innovation	Professionals should document everything in the (medical) file of the child (*parents*)
	A format should be used to guide the conversation with parents in order to obtain consent (*parents/professionals*). This format describes (1) how to conduct this conversation, (2) when this conversation takes place, (3) who is requesting parental consent and (4) who is providing feedback (*professionals*)
	Feedback of the findings of the CDR team should be given to professionals as well as to the parents with the help of a mediator (*parents*). Agreements should be made about who is providing feedback to the professionals and parents (for example the attending physician) and how this is provided to them. Feedback to parents should be provided only in case of individual recommendations (*professionals*). If shortcomings in care are identified, professionals should offer parents their apologies. This was considered important for their grieving process (*parents*)
	When parents are asked to give their consent, they should be informed who is providing them feedback of the findings (*professionals*)
	Professionals, such as the general practitioner, preventive child health care professional or pediatrician, should be approached for information as a standard procedure. The information system of the child can be accessed to see who else is involved in the care of the child/family (*professionals*)
	A guideline/format should be used to help professionals in providing the information needed to review the death. It should be clear how much time the process of information gathering takes (*professionals*)
	Professionals should provide complete and correct information independent from each other to the CDR team (*parents*)
	The benefits of CDR should be emphasized in order to ensure that professionals provide all information to the CDR team (*parents*)
	In case a death is investigated by the Public Prosecutor agreements should be made with the Public Prosecutor/Ministry of Security and Justice for reviewing the death by the CDR team (*professionals*)
	In case of an unexplained death of a child CDR should join the procedure in which these deaths are further examined to clarify the primary cause of death. Data from this procedure can be used for CDR to analyse the death in order to make recommendations directed to prevention (*professionals*)
User	The CDR team should be an independent team in order to prevent bias (i.e., personal interest) (*professionals*)
	The composition of the CDR team depends on the kind of child death that is being reviewed. The chair should be a ‘heavy’ figure who has an overall view and is objective. He/she has the knowledge and has no interest in a particular organisation. Someone from the Health Care Inspectorate could also be considered as a chair, but this could cause some resistance for professionals to cooperate (*p*rofessionals)
	The CDR team is obliged to get at least one preventive activity out of the recommendations made (*professionals*)
	In order to obtain the cooperation of parents to review their child’s death parents should be informed that autopsy data could be used in the CDR (*parents*)
	In order to obtain the cooperation of parents they should fully be informed about CDR by the general practitioner or pediatrician (*parents*)
	Parental consent should be asked a couple of weeks after the death of the child by the pediatrician, general practitioner, preventive child health care professional or just the person who is involved around the time of death. Parents could also be asked whether they like to be requested by the attending physician or somebody else to give their consent (*professionals/parents*)
	In order to obtain the cooperation of parents and professionals data should be anonimyzed at an early stage to conduct a review (*parents/professionals*). To reduce traceability to persons deceased children from another region should be reviewed (*professionals*)
	Parents should have the possibility to check whether the information is correct or not before it is provided to the CDR team. The general practitioner, pediatrician or a confidant could support parents in this (*parents*)
	More publicity to the general public is needed, so that parents know that after the death of their child a review is conducted (*parents*)
Organisation	It should be clear what the implementation of CDR means for organisations (i.e., time investment, costs) (*professionals*)
	The management of organisations should be involved to facilitate time for professionals to cooperate in CDR (*professionals*)
	Consultation with care insurers is needed for financial coverage of CDR (*parents*)
	Collaboration of professionals with the CDR team should be facilitated by organisations (*parents*)
Socio-political context	It should be clear which competencies the CDR team have (*professionals*)
	The CDR process should be adjusted to the Dutch laws and regulations (*professionals*)

## Discussion

In this study we examined the stakeholders’ opinions on the implementation of CDR in the Netherlands.

The identified facilitating and impeding factors are directed at two stages of the process of change, i.e., implementation and continuation. Most determinants were directed at the innovation and user. The relative paucity of determinants associated with the organisation might be caused by the composition of the focus groups that contained mainly participants who have an executive role in the care for the child and family or who are an experience expert as parent of a deceased child. The focus group participants considered the improvement of the quality of (health) care as a benefit of CDR. In the focus groups of professionals more benefits were expected in reviewing groups of certain child deaths. To achieve improvement of (health) care, feedback of the findings of the CDR team to professionals and regional or national authorities, was indicated in the focus groups as necessary. Similar to what has been concluded in the USA and Australia, dissemination of CDR findings to professionals, legislators, agencies and public is one of the important factors to develop a successful CDR program [6, 21]. Reviewing a child’s death was considered in the focus group involving parents as a second opinion that was identified as another benefit of CDR, while in the focus group of professionals the emotional burden was perceived as a drawback of CDR.

The focus group participants perceived the multidisciplinary approach as one of the facilitators to effective implementation. As has been concluded in the evaluation of the CDR process in Australia, the multidisciplinary composition of the CDR team, that is independent of the government, is necessary in order to be effective [21]. Next to this, engagement of motivated professionals and good working relationships are essential for the CDR process to be successful, as highlighted in a study in England [22, 23].

Other facilitators to effective implementation were identified in the cooperation of parents and professionals. Parents were expected to cooperate more easily when they are completely informed about the procedure shortly after the death of their child, oral and written, and are asked for consent a couple of weeks after the death of their child. Signing a consent form was considered to be facilitating in order to obtain the information from professionals. To get the cooperation of professionals the need was stressed to minimize the effort for them and to anonymize data as soon as possible. Using a format for data collection or joining the procedure in case of a sudden and unexpected death was recommended as being helpful in reducing the effort. As found in a study in England, already having structures in place, like a protocol for sudden infant deaths or tools for data collection, could help establish a CDR process [22].

The lack of complete information to review the child’s death was considered to be one of the barriers to effective implementation. Some participants noticed that medical files might not contain all relevant information and expected that professionals might decide not to provide information despite parental consent. Other barriers were the legal consequences for professionals and organisations, time implications, insufficient ratification by the management and professional confidentiality that were perceived in the focus groups involving professionals as well as parents. These barriers were also found in England [22]. Legislation could tackle issues of confidentiality. A legal basis for conducting reviews not only provides the opportunity to share sensitive information and protect confidentiality [6], but it enables also that all aspects of the review process are standardized [7] and protects the independence of the CDR team [21].

The focus group participants recommended to use a consent form and to review a child’s death with anonimyzed data that should reduce issues of confidentiality. They also recommended that the management of organisations should be involved to facilitate time required to cooperate in CDR. If necessary, they recommended to consult medical insurance companies to inform them that CDR is provided as extra care in order to ensure financial coverage. Financial resources are important for a successful implementation of the CDR process [6, 22, 23]. Furthermore, they recommended that the CDR process should be adjusted to the Dutch laws and regulations and agreements should be made with the Public Prosecutor/Ministry of Security and Justice in case a child’s death is investigated by this authority. These recommendations could be used as input for the implementation strategy.

### Strengths and limitation of the study

One major strength of this study is that we could collect data from a very diverse group of professionals of several stakeholder organisations. Another strength was the use of face-to-face focus groups in which the ideas, motives, interests and thoughts of the professionals and parents about the implementation of CDR could be explored thoroughly in a confidential atmosphere [24–26]. The small number of participants in the focus group with parents of a deceased child is a limitation, because we might not have all the opinions and experiences regarding the facilitating and impeding determinants.

Finally, the MIDI proved to be a useful instrument for analysis of the discussions. This framework helped us in structuring the determinants of implementation, thereby increasing the possibility to generalize and make recommendations applicable to other parts of the Netherlands and other countries.

## Conclusion and recommendations

If CDR would be implemented in the Netherlands, which is subject to debate, the determinants identified as facilitating to implementation of CDR and the recommendations provided for the barriers can be used as input for the strategy for implementation. According to the MIDI the focus within this strategy should be particularly on the determinants associated with the user (emphasizing the personal benefits for professionals and parents, the use of a consent form and a format to gather information, and analysing anonymized data), organization (informing managers about CDR) and social-political context (adapting CDR to the Dutch regulations and to the procedures of the Public Prosecutor). In a pilot study it needs to be determined to what extent the chosen implementation strategy is effective and whether the results of reviewing child deaths contribute substantially to achieving the four objectives of CDR. If the pilot shows that CDR is indeed very time consuming and of limited added value in cases in which the cause of death is clear, one might consider to start with reviewing only specific child deaths, such as sleep-related infant deaths or theme-related deaths according to the International Classification of Diseases (ICD). In cases of explained deaths in children a CDR might be conducted only to examine whether family support was provided sufficiently.

## Additional files

10.1186/s13104-016-1966-x Overview of MIDI determinants [1].
10.1186/s13104-016-1966-x Quotes from professionals and parents regarding the facilitators and impeding determinants.
