# Supplementary material for: Stakeholders’ opinions on the implementation of Child Death Review in the Netherlands
Source: BMC Res Notes. 2016 Apr 21;9:228. doi: 10.1186/s13104-016-1966-x (PMC4839131; doi:10.1186/s13104-016-1966-x)
Supplement: Supplementary file 1 — 10.1186/s13104-016-1966-x Overview of MIDI determinants [1]. [file 13104_2016_1966_MOESM1_ESM.docx]

| Appendix 1 Overview of MIDI determinants^[^[^1^](#_ENREF_1)^]^ | |
| --- | --- |
| **Determinants** | **Description of the determinants** |
| *Determinants associated with the innovation* | |
| 1. Procedural clarity | Extent to which the innovation is described in clear steps or procedures |
| 2. Correctness | Degree to which the innovation is based on factually correct knowledge |
| 3. Completeness | Degree to which the activities described in the innovation are complete |
| 4. Complexity | Degree to which implementation of the innovation is complex |
| 5. Compatibility | Degree to which the innovation is compatible with the values and working method in place |
| 6. Observability | Visibility of the outcomes for the user |
| 7. Relevance for client | Degree to which the user believes the innovation is relevant for his/her client |
| *Determinants associated with the adopting person (user)* | |
| 8. Personal benefits/drawbacks | Degree to which using the innovation has (dis)advantage for the users themselves |
| 9. Outcome expectations | Perceived probability and importance of achieving the client objectives as intended by the innovation |
| 10. Personal obligation | Degree to which the innovation fits in with the tasks for which the user feels responsible when doing his/her work |
| 11. Client satisfaction | Degree to which the user expects clients to be satisfied with the innovation |
| 12. Client cooperation | Degree to which the user expects clients to cooperate with the innovation |
| 13. Social support | Support experienced of expected by the user from important social referents relating to the use of the innovation |
| 14. Descriptive norm | Colleagues' observed behaviour; degree to which colleagues us the innovation |
| 15. Subjective norm | The influence of important others on the use of the innovation |
| 16. Self-efficacy | Degree to which the user believes he or she is able to implement the activities involved in the innovation |
| 17. Knowledge | Degree to which the user has the knowledge needed to use the innovation |
| 18. Awareness of content of innovation | Degree to which the user has learnt about the content of the innovation |
| *Determinants associated with the organisation* | |
| 19. Formal ratification by management | Formal ratification of the innovation by management, e.g. by including the use of the innovation in policy documents |
| 20. Replacement when staff leave | Replacement of staff leaving the organisation |
| 21. Staff capacity | Adequate staffing in the department or in the organisation where the innovation is being used |
| 22. Financial resources | Availability of financial resources needed to use the innovation |
| 23. Time available | Amount of time available to use the innovation |
| 24. Material resources and facilities | Presence of materials and other resources or facilities necessary for the use of the innovation as intended (such as equipment, materials or space) |
| 25. Coordinator | The presence of one or more persons responsible for coordinating the implementation of the innovation in the organisation |
| 26. Unsettled organisation | Degree to which there are other changes in progress (organisational or otherwise) that represent obstacles to the process of implementing the innovation |
| 27. Information accessible about innovation use | Accessibility of information about the use of the innovation |
| 28. Performance feedback | Feedback to the user about progress with the innovation process |
| *Determinants associated with the socio-political context* | |
| 29. Legislation and regulations | Degree to which the innovation fits in with existing legislation and regulations established by the competent authorities |

1. Fleuren, M.A., et al., *Towards a measurement instrument for determinants of innovations.* Int J Qual Health Care, 2014. **26**(5): p. 501-10.
