# Supplementary material for: Stakeholders’ opinions on the implementation of Child Death Review in the Netherlands
Source: BMC Res Notes. 2016 Apr 21;9:228. doi: 10.1186/s13104-016-1966-x (PMC4839131; doi:10.1186/s13104-016-1966-x)
Supplement: Supplementary file 2 — 10.1186/s13104-016-1966-x Quotes from professionals and parents regarding the facilitators and impeding determinants. [file 13104_2016_1966_MOESM2_ESM.docx]

Appendix 2 Quotes from professionals and parents regarding the facilitators and impeding determinants

| **Professionals** |  |
| --- | --- |
| **Determinant** | **Facilitators** |
|  |  |
| ***Procedural clarity*** | *It's pleasant for the doctor concerned to know that he/she is the one who will have a conversation with the parents. Then that's clear and that person can keep an eye on the situation.* |
| ***Completeness*** | *I think it's a big advantage that there are so many disciplines around the table, because you hear from different people different things.* |
|  | *I think the complexity should not be a reason not to review a child's death in order to get more clarity. Although it can be very complex in many cases, there might be cases in which a multidisciplinary team discovers circumstances surrounding death or other factors, such as communication problems, from which recommendations could be made directed at a national level.* |
|  | *When I am asked to provide the information we have about the child, I would like to see the information we have is actually provided by us and not by the Child Protection Service.* |
|  | *With regard to the evaluation of the aftercare I think the presence of a behavioural scientist as one of the core members of the CDR team is positive.* |
| ***Compatibility*** | *What I've read about it, is that the perinatal audit and CDR in England are actually pretty much the same only just a different target group.* |
| ***Relevance for client*** | *A strength of the method is the fact that a review is not only conducted in cases of special circumstances, but in every child death. CDR is offered to every parent of a deceased child. So, it is not assumed that suspicious circumstances had been present leading to death.* |
|  | *It must be taken into consideration how stressful it is for the family and also for (health) care professionals. But I think you can limit the burden. If you organize it well then I think it should be acceptable and then I do see the benefits.* |
|  | *I think a very extensive investigation has an added value in addition to the NODO procedure. If you gather information from other disciplines more data come to light than just only a home visit and a postmortem examination performed by the pathologist after the death of a child.* |
|  | *For the improvement of the analysis of individual cases I do not think you need a CDR, only for larger groups of child deaths.* |
|  | *I think you should want to know why children die in the Netherlands.* |
|  | *Unless the communication does not work well within the group (i.e. of professionals), the group will never reveal their problems. In that case it is good to look at the case from the outside with a helicopter view.* |
|  | *I think it shows some respect to the child whose death is being investigated seriously.* |
|  | *Depending on the situation support of parents is important. We are focused, of course, in the first place on the safety of the other child.* |
|  | *Quite often the police doesn't hear from the hospital what has happened. The police must therefore get the information herself. By providing feedback you know what took place, what went well or wrong, children in problem situations or other children present in the family ....* |
| ***Personal benefits/drawbacks*** | *It may be a good tool to check if you've done well.* |
|  | *Anonymize information as soon as possible, because of the confidentiality of data obtained.* |
|  | *Positive is the fact that you are immediately aware of reporting every child death to the CDR team, and that you have to report every death, which is not an automatic procedure* |
|  | *Eventually, in the process of dealing with the death of their child it could be helpful for parents to know where their child has died from. Even if nothing has been found, there has been made some effort in considering factors that might have contributed to the death.* |
|  | *In many situations I have found it very pleasant that feedback is given.* |
| ***Outcome expectations*** | *When a child's death is discussed within the CDR team, a conclusion could be that in spite of all the possibilities out there and despite the fact that there are a lot of agencies involved, it is not possible to obtain a closed system to prevent the death. Then you want this kind of cases to be aggregated to a higher level, so you can prevent similar cases.* |
|  | *How are those professionals trained? This could be questioned when a CDR team discusses a deceased child. That's an improvement.* |
|  | *You can learn from deceased children for future cases, I think.* |
|  | *There are of course many examples where terminal care could be improved. I think it is possible and important to do that.* |
|  | *I think that you can recognize patterns if groups of child deaths are combined from which lessons can be learned.* |
|  | *I see potential improvements in health care by implementing CDR.* |
|  | *Not to point to the past, but to get advice for prevention in the future.* |
|  | *I really think that children, especially the small ones, die of abuse that we have never recognized. You can mistreat a child without seeing it.* |
|  | *But I see it much more in a broader context in which in the Netherlands there will be more attention for prevention.* |
|  | *Child Death Reviews seem to me to be a very good preventive tool for the future not only to professionals but also to the parents if more is known about causes of death. It's just a taboo to talk about the causes of death of children.* |
|  | *A great strength of CDR is that you may be able to recognize patterns by bundling cases. I think that's very important.* |
|  | *I still think that CDR has an added value. Not to shake one's finger at the organization, but in order to detect patterns and to make recommendations.* |
|  | *Trends could be discovered that can be translated into national policy.* |
|  | *Substandard factors in care within certain hospitals could be noticed.* |
|  | *Recommendations from different angles. I think that's important.* |
|  | *And to check whether there is after-care.* |
|  | *If something happened with a child and doctors will evaluate it’s death with recommendations directed at a regional level, then I have a positive attitude towards CDR.* |
| ***Client cooperation*** | *When parental consent is not asked shortly after the death of their child you (as professional) have time to think about how to formulate it. You also have more time to gather your information and give the information to parents. I think the chance of success (i.e. participation of parents) is much greater.* |
|  | *Well, parents also want to know what has happened to their child who died.* |
|  | *We assume that parents are well informed about the purpose of CDR.* |
| ***Descriptive norm*** | *I think some pediatricians are highly motivated.* |
| ***Formal ratification by management*** | *Perhaps managers could play a role for example in the pledge of secrecy.* |
| ***Legislation and regulations*** | *If parental consent is present, confidentiality is not a problem.* |
|  | *The objective of a CDR team is not to evaluate the individual case completely whether procedures went well, but more to aggregate to a higher level.* |
|  | *If parents gave their consent, confidentiality is not a problem anymore.* |
|  | *If data are anonimyzed then it can be used for research.* |
|  | *If parents gave their consent, information could be provided to the CDR team.* |
|  | *If the objective of CDR is to investigate the causes of death and what conditions are needed to prevent child deaths in the future than it is allright to use anonymous data.* |
|  |  |
|  | **Impeding determinants** |
|  |  |
| ***Procedural clarity*** | *For me the stratification in that process is still a bit unclear.* |
|  | *I agree with providing feedback, otherwise open ends remain present with the consequence that professionals actually do not notify a child's death anymore.* |
| ***Completeness*** | *But in order to be able to aggregate the conclusions of a CDR team to a higher level, I wonder whether a CDR team, regardless of the expertise of its members, gets its own wisdom to make some reccommendations to other hospitals.* |
|  | *It seems difficult to get data from paper (medical) files when no interviews with the people involved are held.* |
|  | *What surprises me a bit is that professionals involved are not present during the CDR meeting. I think that's remarkable, because that will make the communication more equally clear and obvious. Written information could be misinterpreted.* |
|  | *You should also consider that some people are not going to give the information. What is the quality of a CDR if you have only half of the information? Does it make sense to discuss it?* |
|  | *The data collection is not so simple. Where do you draw the boundaries of what you can not automatically check? Especially when you think that there might be an important psychological factor that has contributed to the death of the child. You need to collect a lot of data if you want to avoid to be biased.* |
|  | *Not everyone has an extensive (medical) file, but one can have a lot of experience with the parents or deceased child that is not noted in the (medical) file.* |
|  | *If parents give their consent, it doesn't mean that doctors provide information.* |
|  | *If you do not ask parental consent to request that information, it is still questionable whether you will receive the information from the doctor or other instances where the child is known.* |
|  | *But the strictly forensic group: the parent who beats his child to death, is under investigation of the Public Prosecutor and you really do not get the information until the criminal case has been closed. But I'm sure you're not going to get that information, because a Public Prosecutor just has a duty of confidentiality.* |
|  | *Each region is organized very differently. In our region the reference index will soon be used. VIS 2 (registration system) tells me basically nothing. That makes it difficult, because everything is organized so differently everywhere.* |
| ***Complexity*** | *The complexity is found in the detailed description of the situation. It requires an accurate report from obstetricians and pediatricians in order to figure out what exactly has happened.* |
| **Relevance for the client** | *But what is the added value in case a child is dying from leukemia in which the disease course is clear? You may assume that all procedures have been properly completed. Can you then still learn from that case?* |
|  | *I personally think the aim is very good, but not for the individual improvement. In that case a simple and faster way could be used.* |
|  | *But can you then give feedback to the parents? That is more difficult.* |
|  | *For me cooperation depends on the idea of how burdensome it is for that family who has just lost a young child.* |
|  | *And you also want to prevent some medical difference between the CDR team and board of medical examiners. Suppose the CDR team has come up with wrong conclusions afterwards. That seems to me a tragedy for those parents who experience a trauma already because of the death of their child.* |
| ***Personal benefits/drawbacks*** | *If data are not protected in the right way, it will give the blockage in providing information.* |
|  | *Protection for themselves (i.e. professionals) and for the organizations.* |
|  | *The difficulty is that there is a committee that examines what, if any, could have been improved in a case. In essence it means for those parents that there has been a mistake somewhere. Regardless of the utmost care with which such a committee is trying to define conclusions and recommendations, it may have the wrong effect in individual cases. Then people feel threatened.* |
|  | *It applies not only to doctors but to all those who provide information. What is equally important is the area of tension. The point is not who is right, but there will be professionals who are afraid to make their data available because they might even doubt themselves.* |
|  | *At that point the doors are closed and you say nothing. Parents could also have the same experience, because they are considered guilty until proven otherwise. There is always a double meaning. I do not know if people realize this well enough.* |
|  | *I totally agree with you. Not that I am not willing to cooperate. Indeed, it will be discussed although it is not the objective of CDR.* |
|  | *As a doctor you need to provide your information to an independent committee that analyses the case. You do not know how independent those people are. That simply plays a role.* |
|  | *I still find it difficult to obtain the information from the CDR team while I did not participate in the discussion. In giving feedback of the findings to parents I may get questions about which I do not know the motivation that well.* |
|  | *If you are involved in such a case, especially if you're directly involved, it will cost you emotionally and practically very much time. Then the paperwork is not that what everyone is waiting for.* |
|  | *It is emotionally stressful and it takes an incredible amount of time.* |
|  | *It is also difficult to decide what you want to know. On the one hand you have to be very broad and, on the other hand, it must be considered that the person from whom you request the information has got minimal extra work.* |
|  | *Anonymity of the practitioner and the patient.* |
|  | *Anyway, if it's something special, then of course everyone knows what it is about.* |
|  | *It is quite a lot of work if you want to review all deceased children.* |
|  | *But there is also a lot of coordination in the retrieval of that data. That's a lot of work.* |
|  | *I think that professionals might feel assaulted if there is something new, because as a professional you think: "I have that conversation with the parents and I do it very well."* |
| ***Outcome expectations*** | *I find it difficult to look for the tangible advantages of CDR.* |
|  | *And you will not learn from every case, I think, except the small improvements at the individual level of course.* |
|  | *Before you know you obviously have an incredible amount of different focus points where you could do something with it, but what can not all be accomplished.* |
|  | *It seems to me terribly difficult for a committee with all kinds of different disciplines to have an opinion at a given time. So I'm wondering what you can acchieve with the natural causes of death.* |
|  | *You actually need enough cases with question marks to establish the value of CDR.* |
|  | *On the other hand I wonder whether CDR is the right method. Could it be done on a smaller scale? There are consultations that are probably not optimal.* |
|  | *Parents obviously want to know the cause of their child's death. So I do not understand the aspect of confidentiality. If the Health Care Inspectorate is not present during the case discussion, then professionals can freely talk about the deceased child.* |
| ***Client cooperation*** | *The barrier in the cooperation in order to provide data are the legal consequences. Parents might easily contact a lawyer when they think that something went wrong in the provision of care.* |
|  | *Negative publicity in the media makes people defensive.* |
|  | *Even something like this. If you even want to interview parents and they have a very negative experience with professionals.* |
|  | *Suppose parents know more about the cause of their child's death, they might decide not to participate.* |
|  | *If parental consent is asked at the moment their child has died, I think the threshold for participation for some people becomes higher.* |
|  | *And if you discuss it with your team, then there is always something that could have been done better and that keeps you busy. This limits you in giving space to others who are evaluating the case.* |
|  | *If you ask me to obtain parental consent on day 3 after CPR failed, at that moment I am still thinking: "What have I done wrong that this healthy child has died?" Then I am a little less approachable for asking parental consent than after 6 weeks thinking that it had to happen in this way.* |
|  | *If it creates more work than the threshold to participate is much higher.* |
|  | *Parents may refuse to cooperatie or doctors can think: "I do not support CDR. I'm not going to put more pressure on those parents." This also plays a role.* |
|  | *It could also be that dad wants to participate, but mom does not or vice versa. This is also seen in suspicious cases of child abuse.* |
|  | *There was a recent piece in the newspaper about parents who lost a twin at 16 weeks. I was then accidentally involved from the beginning to the end. The information was not valid on many points.* |
|  | *It is also important to make clear agreements about who is then informed. I think that is a precondition. It is not effective when the CDR team members go apart after such a meeting and no agreements are made about follow up.* |
|  | *What I always find a pitfall if proper arrangements are made with people is that parents seem to cooperate. They give you the impression that they cooperate, but in fact they don't.* |
| ***Descriptive norm*** | *I think when you talk about the burden for pediatricians support from all paediatricians in the region is necessary. I think that is not so easy.* |
| ***Knowledge*** | *The entire medical procedure that has preceded death will be examined. I think that's not so easy.* |
|  | *It is a very extensive procedure to evaluate medical practice, which of course happens sometimes especially when parents think that mistakes were made. A regional or national disciplinary committee reaches a decision with many remarks after a lot of hours of work. With many caveats often. It will be difficult to review child deaths in the Netherlands and to draw lessons from it.* |
| ***Time available*** | *I think that time plays definitely a role.* |
|  | *It is emotionally stressful and it takes an incredible amount of time.* |
|  | *It is difficult to make a paper file anonymous. That is quite a job.* |
|  | *Those who must provide the information, are all people who are very busy.* |
| ***Legislation and regulations*** | *Confidentiality* |
|  | *Anonymity of the practitioner and the patient.* |
|  | *In the interest of the investigation which is still ongoing, a forensic physician just can't give information merely because he/she can only report to the Public Prosecutor.* |
|  | *Parents can not be forced to participate.* |
|  | *It should be figured out how CDR fits well into the Dutch system of health care and justice that is a totally different culture in relation to other countries where CDR is implemented.* |
|  | *The CDR procedure as used in England and USA can not be implemented in the Netherlands. It should be translated into a Dutch version.* |
|  | *Information could not be requested without the signature of the parents.* |
|  |  |
| **Parents** | **Facilitators** |
|  |  |
| ***Completeness*** | *If all disciplines provide information, the chance will increase to come to the proper reconstruction about what exactly happened. When you hear that from one perspective, it is always coloured and contains miscommunication and occupational deformation.* |
|  | *What I find very pleasant is that all care providers must provide information to the CDR team.* |
|  | *The chances are that nothing is being ignored because of the independent input.* |
|  | *They (i.e. professonionals) can learn from each other.* |
| ***Relevance for the client*** | *That they might say: "We are professionaly considering that as very pleasant, because that is something we have noticed very few."* |
|  | *The quality of care provided in a hospital, can thus be improved. It is a process in which we continually try to improve quality.* |
| ***Personal benefits/drawbacks*** | *A major advantage of this procedure for parents is that signing a consent form stresses the respect towards parents who have just lost a child. I think that it is very important for a parent that he/she will be acknowledged and knows that the death of his/her child will be examined thoroughly. Maybe there are some recommendations to prevent a similar death.* |
|  | *The information is provided to the CDR team after which the child's death is examined again. If something went wrong, the parent can ask the doctor to explain better what has happend. I think that in itself is a very pleasant idea.* |
|  | *For parents it can be important to independently verify what has happened with their child. It is not only for the future and for other children, but also for parents who are offered an independent second opinion.* |
|  | *Parents who have lost a child, think differently about the value of a child. I notice it myself. I keep more of the living children for myself, but I also think for the children to show them something of their youth. This can also be an advantage for parents that their child's death is properly analyzed.* |
|  | *I think it (CDR) gives more involvement.* |
|  | *I think that professional associations also receive information from which they can benefit.* |
|  | *That is a common experience that can be very comforting for parents because the name of the child may be mentioned.* |
| ***Outcome expectations*** | *Exactly, I also hope that this might be the interpretation in our society, in which we actually keep the deceased almost quiet.* |
|  | *And as a professional you do not want to condemn. You try to develop guidelines to improve communication and training of professionals who just started.* |
|  | *CDR has the advantage to provide parents prevention in their future health. It is known that the relationship between parents comes under pressure when they have lost their child, because men and women grief differently. If CDR is properly arranged, it will provide long-term prevention in terms of health and retaining work.* |
|  | *You can view this in two ways. A CDR is only for the future and for improvement, but not for the individual case. So it's anonymous and parents are not informed. It is not about making mistakes and liability. Or data are not anonymized and retrievable for the parents. This is of course a big difference.* |
|  | *I do not know how you should solve this legally, but CDR seems to me for the future, the science and improvement and not for the claim culture in the individual case.* |
|  | *I hope they also conclude that when things are not optimally proceeded that it can be improved.* |
|  | *I think that a CDR team has more authority to provide good advice to the government that does not depend on an individual hospital or local situation.* |
|  | *Trends can be identified.* |
|  | *More co-operation between professionals.* |
|  | *I think CDR has also an advantage in making recommendations for the future that put more weight on the scale when a pattern is identified by the CDR team. As a parent you do not get all the information by yourself.* |
|  | *Maybe CDR might result in training for assistants in this subject or being informed about it.* |
|  | *Perhaps CDR might even provide input for peer review within professional groups.* |
| ***Client cooperation*** | *It is nice that you can still consult someone like the general practitioner. Parental consent is requested later.* |
|  | *I thought that the decision for that signature follows immediately after the death, but parental consent is given later. Then you're more likely that parents want to participate.* |
|  | *I suppose it's all been communicated to parents and that they sign a consent form.* |
|  | *It is also important that parents clearly are informed about the procedure to which they give their consent.* |
|  | *As a parent you have lost a child that is very special, but at the same time you can do something positive. By cooperating in CDR parents could contribute to the prevention of future deaths* |
|  | *That may be an argument to winn someone over.* |
|  | *I think this can be illuminated from two sides. Maybe you do not want it at first, but later you do want to participate in CDR.* |
|  | *You need the parents to get the information, so you also owe them something (i.e. feedback of the findings).* |
|  | *Starting with respect and appreciation. Positive reinforcement towards professionals is very important.* |
|  | *It seems to me useful to mention positive points of CDR in an annual report.* |
|  | *You could also think of people who have the gift of writing good articles. They could describe a CDR case in a professional journal.* |
|  | *If CDR is well-known people might approach it positively.* |
| ***Legislation and regulations*** | *I suppose it's all been communicated to parents and that they sign a consent form.* |
|  | *It is also important that parents clearly are informed about the procedure to which they give their consent.* |
|  |  |
|  | **Impeding determinants** |
|  |  |
| ***Completeness*** | *The conversation with the pediatrician that takes place 6-8 weeks after the death needs to be reported which is not the case.* |
|  | *There is no conversation with the parents before the information written down on paper is provided to the CDR team.* |
|  | *The input of information has to be recorded. But if the information is not written down, how do you deal with that?* |
|  | *I am thinking about the first responders who have to write the reports and provide these to the CDR team.The fact that a general practitioner (GP) may think that he/she should actually make a home visit after the first phone call, which he/she did not. The GP does not include that information in his/her report.* |
|  | *As a professional you have a conversation with another professional or you get information on paper that can result in miscommunication.* |
|  | *Thers is a disadvantage of documenting electronic data.* |
| ***Complexity*** | *But maybe they (i.e. professionals) initially think "more work and more bureaucracy."* |
| ***Personal benefits/drawbacks*** | *In my opinion you give away your child for the second time. It's taken from you and you want to have and keep everything. Then the question is asked to further analyze your child's death and you give away your child again.* |
|  | *Parents are very willing to eventually respect those mistakes. They are much less willing when they feel there is not full disclosure.* |
|  | *People are all producing cows, so they think of more work. One thinks that he is doing well, so why do I have to provide that information? The other thinks he might not do so well and they're going to check him.* |
|  | *They (i.e. professionals) will think: "let me do my job".* |
|  | *There will be parents who figured out that there is a communication problem.* |
|  | *Parents need to know that a team is talking about their child and they are waiting for the conclusion. As a parent you can be suspicious about that because a parent might think that professionals protect themselves.* |
| ***Outcome expectations*** | *It should not get the title of "we're going to find the ones who are guilty." This will lead to a legal context which is not the objective of CDR.* |
| ***Client cooperation*** | *Otherwise, I expect that you are going to get a lot of problems in obtaining the signature on the consent form. That seems to me a very difficult job. I guess I had not signed the consent form.* |
|  | *I think that if parental consent for autopsy is asked in a blunt manner or by a wrong person at the wrong time and as a parent you have said 'no', then this will determine the further course of the investigation.* |
|  | *That is almost a taboo for a parent to indicate that there is no permission for autopsy.* |
|  | *You might even think that the forensic doctors could also be questioned who is going to ask parental consent.* |
|  | *You have to take the media with you, otherwise they can manipulate the information.* |
|  | *I thought right away that parental consent should not be asked by a person, who you do not know. You easily think that he/she has a personal interest and you do not want that person asking for consent.* |
|  | *Sometimes it is the chemistry with the person who is asking parental consent which can be very important.* |
| ***Formal ratification by management*** | *When actions should be set out within certain professional groups that are employed, you have to do with a management that must support these actions and has to give time to be able to implement them in practice* |
| ***Time available*** | *Professionals could say: "I have no time for writing a report."* |
|  | *Management might provide not enough time to provide information.* |
